# Leçons Orales, &c.—Clinical Lectures in Surgery, Delivered at the Hôtel Dieu, Paris

**Published:** 1833-04-01

**Authors:** 


### Medico-Chirurgical Review,

### No. XXXVI.

JANUARY 1 TO APRIL 1, 1833.

### I.

LEÇONS ORALES DE CLINIQUE CHIRURGICALE, FAITES A L'HÔTEL DIEU DE PARIS. Par *M. le Baron Dupuytren*, Chirurgien en Chef. Recueillies et publiées par une Société de Médecins. —(Clinical Lectures on Surgery, delivered at the Hôtel Dieu at Paris. By Baron Dupuytren.) Tome I. Paris, 1832.

A COMPARISON of modern medical literature with that which has preceded it, exhibits the most striking difference in the preponderance of facts over opinions and theories. That a disrelish for the latter, and consequent taste for the former, is increasing, not only among medical readers, but in society in general, is equally evident. Intelligible facts will alone sell, whether in the form of Penny Magazines, or in treatises written by Brewsters or Babbages. The few medical works which have escaped the destroying hands of trunk-makers, or the conversion into "shrouds for pilchards and red herrings," are those alone which have contained a large preponderance of facts. The few theories in which Hippocrates indulged, or the occasional opinions of Sydenham as to the proximate causes of diseases, are rescued from the oblivion to which they would have been necessarily consigned from their absurdity, only by their being appended to practical remarks which will ever continue valuable, as the expression of facts discovered by close observation. The description of diseases by Celsus and Aretæus are pregnant with instruction, and will last as long as medicine continues to be studied; but who now, except for amusement, or as a matter of history, peruses the wild theories of Paracelsus, or many of the more modern medical vagaries of Darwin or Beddoes. Not that such imaginative men of genius are without their ultimate use, by setting inferior intellects at work in channels which they would never have beaten out for themselves. This increased attention to the importance of facts in studying disease cannot be promoted more effectually, than by clinical instruction, fully carried into effect as it is in the French hospitals. No plan is more adapted for testing the merits of the doctrines of the Professor, or of estimating the advantages of the treatment he pursues. The value of any mode of practice, as laid down in lectures, can alone be tested by experiment; in clinical instruction, the principles are illustrated by actual practice, and the vacant bed, the " dead-house" table, the distorted limb, or unexpected post-mortem appearances, are facts which cannot be hidden, and which, on a large scale, can never be set aside by specious reasonings. That this teaching by example, and illustrating general principles by actual cases, is the mode of all others the most calculated to make a permanent impression on the mind of the student, few will be disposed to deny. We were, therefore, glad to see the announcement of a work purporting to be the clinical lectures of Baron Dupuytren, who, as a professor of clinical surgery to the School of Medicine in Paris, stands without a rival. The printed lectures have not the sanction of M. Dupuytren's name, but, as the most important cases have been furnished by MM. les Docteurs Marx, Paillard, and Fournier, pupils and friends of the Baron, it is not probable that they are published without his approbation. Considerable difficulty must have been experienced by the reporters in the compilation of these lectures, from the disjointed way in which they are frequently delivered. The report of the progress of the most remarkable cases, with the observations it may suggest, being given daily. If M. Dupuytren has in his wards many similar cases of particular interest, he makes them the excuse for a surgical disquisition on the peculiar disease; if this requires a lengthened discussion, he resumes it daily, after giving the report of the cases under treatment. Thus, in process of time, his clinical lectures embrace every topic in surgery. Two thick octavo volumes are just published, containing these lectures, with a promise that the subject will be continued. The plan which M. Dupuytren adopts, in order to arrange the arguments on both sides, or the diagnostic signs in the clearest manner, for the instruction of his class, by putting questions which he himself answers, has not been interfered with. These volumes must, therefore, contain much elementary matter, which would be deemed superfluous in a written treatise; in the analysis which we intend to give, we shall of course omit all such particulars, and, by pruning and compressing, we shall endeavour to put our readers in possession of all that is new or valuable.

### ART. I.—ON PERMANENT RETRACTION OF THE FINGERS.

The cause of this peculiar retraction of the fingers, and particularly of the ring fingers, has been attributed by different authors to rheumatism, gout, external violence, fracture—to the effects of inflammation of the tendons of the flexor muscles, or to a species of anchylosis. Boyer, in his Traité des Maladies Chirurgicales, denominates it crispatura tendinum, but describes it very superficially. It affects those individuals whose occupation causes them to make a point-d'appui of the palm of the hand. Thus, it has been observed in coachmen, who are obliged constantly to use the whip—in keepers of wine-shops, who are continually tapping their wine-casks—in masons, who grasp stones with the extremities of their fingers—in farmers, and, in one instance, in a man with a large correspondence, who took particular pains in sealing his letters. We remember to have seen it in both the hands of an inferior officer in the revenue service, who had been accustomed to steer, for a long series of years, the Custom-house boats, and, consequently, to hold the ropes continually in the palms of each hand. It commences by the individual perceiving that he can extend with less ease the fingers of the affected hand; the first phalanx of the ring finger is first retracted, but, at this time, its palmar surface presents no nodosity, its two last phalanges are straight and moveable, although the first is bent at a greater or less angle, and cannot be rendered straight by the most violent force. One person, in order to cure this, lifted with the finger different weights, until he supported 150 ℔s. without extending the phalanx. When the ring finger is very considerably bent, the skin is thrown into folds, whose concavities are towards the fingers, and convexities towards the radio-carpal articulation; this is owing to the natural adherence of the skin with the diseased parts beneath, and not to any disease of the skin itself, which dissection proves is unaffected. On touching the palmar surface of the ring finger, a tense cord is felt, extending from the top of the finger to the superior extremity of the palm of the hand; this almost entirely disappears on bending the finger. On endeavouring to extend the finger, it will be seen that the tendon of the palmaris brevis is put in action, and that this motion is extended to the superior part of the palmar aponeurosis; the continuity of these parts explains their simultaneous action. At this period, the adjoining fingers cannot be completely extended, the patient can grasp only small substances, and, if he squeezes them strongly, he feels acute pain; whilst his hand remains at rest, there is no pain, but it comes on if he extends his fingers forcibly. M. Dupuytren has been consulted in upwards of thirty or forty cases, and he has been in the habit, in his clinical lectures, of bringing forward the opinions of a crowd of authors, as to the nature of the retraction; these, or the arguments against them, we shall not here enter into, but proceed at once to the detail of the dissection, which explained to the Baron the cause of the deformity, which had not hitherto been suspected.

M. Dupuytren having obtained the arm of a man who had just died, and whom he knew was subject to this affection, commenced the dissection by taking off the whole of the skin from the palm of the hand, and the palmar surfaces of the fingers; the folds and wrinkles entirely disappeared, shewing clearly that the disease was not situated in the skin itself. On exposing the palmar aponeurosis, it was found stretched, retracted, and diminished in length; from its inferior part proceeded the kind of cords which were attached to the sides of the affected finger. On endeavouring to extend the fingers, the aponeurosis became tense and crackled; on cutting the prolongations of it, which were attached to the sides of the fingers, the contraction ceased, the fingers returned to their usual state of demi-flexion, and the least effort completely extended them. In order to leave no part unexamined which might be implicated in this affection, M. Dupuytren exposed the tendons, and found their size, their mobility, and their polished surfaces perfectly natural; as well as the bones, the ligaments, and the joints themselves, in a completely normal state; he was then justified in concluding that the contraction was owing to an unnatural tension of the palmar aponeurosis, produced by a contusion of the part, the consequence of the too strong and too prolonged action of a hard body in the palm of the hand. The diversity of opinions as to the cause of this deformity has necessarily produced much uncertainty in the employment of therapeutic agents. M. Bennati, in consulting Sir A. Cooper as to the case of an Italian, named Ferrari, who was subject to this retraction, was told by him that it was incurable. Such is also the opinion of many other surgeons. M. Dupuytren had tried fumigations of emollient vapours, cataplasms, leeches, mercurial frictions, douche baths of every sort and temperature, without success. In one case he tried permanent extension by a machine contrived for the purpose, but the pain it produced was so severe that he abandoned it. The flexor tendons have been divided in two instances, but to no purpose; and in one case the patient's life was endangered by the inflammation extending along the sheath of the tendons. Shortly after these operations M. Dupuytren was consulted in the following case.

In 1811, M. L., a wine merchant of Paris, having received a stock of wine from the South, assisted his workmen in arranging the casks one upon another in his cellars. In raising a very heavy cask by placing his left hand beneath its prominent edge, formed by the ends of the staves, he felt a cracking, and a slight pain in the palm of the hand. Increased sensibility and stiffness remained for some time, but these symptoms gradually disappeared. The accident was almost forgotten, when he observed a slight retraction of the ring-finger towards the palm of the hand, so that he was unable to extend it as much as the others. There was no pain, and he neglected the slight deformity, which every year became more marked. At the commencement of the year 1831, the little and ring fingers were completely flexed, and applied to the palm of the hand; the second phalanx was folded on the first, and the extremity of the third touched the middle of the cubital border of the palmar surface. The skin was thrown into folds, and drawn towards the base of the two retracted fingers. M. L. now consulted several practitioners, who recommended the division of one or both of the flexor tendons; subsequently M. Dupuytren was consulted, who immediately stated that the disease was not situated in the tendons, but in the palmar aponeurosis, a division of which would produce a free motion of the fingers. The operation was thus performed. The hand being firmly fixed, M. Dupuytren commenced by making a transverse incision, of ten lines in length, opposite the articulation of the phalanx of the ring finger with its metacarpal bone; the bistoury divided at first the skin, and next the palmar aponeurosis, with an audible cracking sound; the finger immediately straightened itself, and could be extended with almost as much ease as when in the natural state. Wishing to avoid the pain of a new incision, the operator endeavoured to continue the division of the aponeurosis by introducing the bistoury in a transverse direction, deeply beneath the skin of the side of the cubital border of the hand, in order to liberate the little finger, but in vain. He then made a transverse incision opposite the articulation of the first and of the second phalanx of the little finger, and thus detached its extremity from the palm of the hand, but the rest of the finger remained firmly flexed. The skin and aponeurosis was next divided by an incision opposite the articulation of the phalanx with the metacarpal bone; this produced a partial but not a complete restoration of the flexibility of the part. A last transverse incision was then made opposite the middle of the first phalanx itself, and this must have divided the insertion of the palmar aponeurosis, as the little finger could immediately be extended with the greatest facility. The incisions, which bled but little, were dressed with dry charpie, and the little and ring fingers were extended by means of an apparatus fixed to the back of the hand. The day of the operation and the following night were passed with little or no pain. During the next day the hand became slightly swollen, owing to the clumsiness of the instrument. Another was then substituted, consisting of a half-cylinder of pasteboard, terminated by four metal rods, which could be lengthened or shortened at pleasure, and which were surmounted with a kind of thimbles to embrace the ends of the fingers. The patient found at first some relief, but in the evening the pain and swelling returned. He was ordered to have his hand sprinkled constantly with a solution of acetate of lead in cold water, without removing the apparatus. This diminished the pain and swelling. Without giving the daily report of the progress of the case, it will be sufficient to state that suppuration became established, and the wounds, at the end of about twenty days, were completely cicatrized. The machine was used for more than a month, and when it was removed the fingers were found, with the exception of the temporary stiffness, occasioned by continual extension, to be so flexible as to allow the patient to bend them easily. A month after this M. L. only employed the machine during the night, and the articulations were becoming so supple as to show that the tendons remained healthy, and that in process of time the motions of the fingers would be completely re-established.

M. Dupuytren repeated this operation Dec. 5th, on a coachman, aged 40, who applied to him for the relief of the deformity which existed in both hands. When he came to the hospital his fingers were so bent that they were not more than an inch and a half distant from the palm of the hand, the skin of the palm forming folds whose concavity was turned towards the fingers. On extending the phalanges, the cords passing from the fingers to the palm were very evident. The patient being seated, M. Dupuytren held the right hand, and made with a bistoury two semi-circular incisions, the one at the base of the ring finger, in order to divide the two digital prolongations of the palmar aponeurosis which were attached to this finger, and the other an inch and a quarter beneath the former in the palm of the hand, to make a second section of this digital prolongation, and to separate it by its base, from the body of the palmar aponeurosis. The ring finger was immediately restored to its normal position. The patient becoming very weak, the operation on the left hand was deferred until another day. The hand was dressed in the same manner as was adopted in the preceding case, and the result will most probably be similar.

Although three facts are not sufficient to establish a general doctrine, yet, by awakening the attention of surgeons, they may be of considerable benefit to science, by multiplying the observations on the causes, signs, effects, and treatment of this disease, and chiefly in the operation which has here been proposed and adopted. In conclusion, M. Dupuytren recommends caution, lest a false application of these observations may be made, by supposing that all retractions of the fingers resemble these, and require the same operation. In a subsequent clinical lecture (ART. XVII.) this subject is resumed, as there were several cases in the hospital illustrating the different species of retraction of the finger.

*1st Case.* A boy, aged 14, admitted under M. Sanson for a white swelling of the ankle-joint; has also retraction of the little finger of the left hand, of very long standing. There is not the least motion in the articulations of the phalanges with each other, but the junction of the first with the metacarpal bone is perfectly free; on bending the finger backwards there is no palmar cord; hence the disease is in the articulations of the phalanges.

*2d Case.* In this individual the two last fingers are constantly bent towards the palm of the hand, but they are easily extended, and there is no cord. This is owing to a sabre-wound on the back of the hand having divided the two extensor tendons. A contused wound may produce a similar effect.

*3d Case.* In this patient the little finger is curved into an arc of a circle, the articulations are unaffected, there is no palmar cord, and the tendons of the flexors and extensors are sound. The retraction depends on a disease of the skin which has destroyed its palmar surface to a great extent: this was owing to a contused wound from the wheel of a carriage. The wound was cicatrized by placing the edges in contact, not by the production of a new cutaneous tissue;—a narrow cicatrix and retraction of the finger was the consequence.

Burns of the palmar surface improperly treated by neglecting to extend the fingers, is a frequent cause of their contraction. But in these cases there are no prominent hard cords extending from the fingers to the palm. Retraction from deformity of the articular surfaces of the phalanges, occasioned by certain occupations, is not uncommon. Women who constantly knit, and who are consequently obliged to retain the little finger separate from the others, and strongly bent during a very long time, in order to support the thread they employ, have often a retraction of this finger from deformity of the corresponding articular surfaces of the first and second and second and third phalanges. This was very common in France, and is still so in Germany, where the women of Berlin and Dresden knit whilst walking.

*4th Case.* A strong young woman, a lace-maker, has the four last fingers of each hand retracted towards the palm, forming the fourth of a circle; the articulations of the phalanges with the metacarpal bones are free. The fingers may be bent backwards; this shows no cord. The second phalanges however cannot be bent backwards on the first, owing to a deformity of their articular surfaces, produced by the occupation of the patient.

*5th Case.* This patient is a tailor; such persons are obliged to keep the fingers of the right hand constantly bent. In this instance it is impossible to straighten the ring finger, and such attempts produce pain, but nothing indicates disease in the palmar surface. The retraction depends on the presence of a ganglion, or accidental serous kyst, at the articulation of the first with the second phalanx. Wounds of the flexor tendons may be mistaken for this retraction, but the prominence of the cord formed by the aponeurosis is much more superficial, nor does it yield to extension; whilst in extending the finger contracted by a divided flexor tendon, the tendon of the palmaris brevis is depressed, and the prominence almost entirely disappears.

*6th Case.* The middle finger of this patient is curved into half an arc of a circle; from its pulp a cicatrix passes to its border, in which is felt a round and hard cord, which is the tendon. This displacement and retraction was produced by the surgeon having opened the sheath of the tendon, in making an incision for a whitloe.

*7th Case.* This patient has the palmar surface of the third phalanx strongly bent on the second, of the indicator finger of the right hand. There is complete anchylosis of the articulation. A wound on the dorsal surface had penetrated the joint, and inflammation, with its usual consequences, followed.

*9th Case.* A ball from a pistol passed through the fore-arm of this patient, without fracturing the bones. The cubital nerve was divided, and paralysis of the internal side of the arm, and two last fingers, followed. M. Dupuytren enlarged the wound immediately, simple dressings were applied, and the wound cicatrized in the course of a month; but the paralysis remained, as well as retraction of the two fingers. The articulations are free, but, on our endeavouring to extend the fingers, there is much resistance and pain at the cicatrix. This is produced by the loss of substance which the flexor muscles have sustained.

This lecture which is especially valuable, from its clearly proving the nature of an affection hitherto unsuspected, illustrates the clear and methodical manner in which M. Dupuytren arranges his cases for the elucidation of his subject.

### Art. II.—On Cataract.

In following the plan we have laid out, of only placing before our readers the peculiar views of M. Dupuytren, or striking and instructive facts, illustrating modes of treatment which are commonly adopted, we shall pass over the descriptions of the various forms of cataract, which are perhaps better described, and, we cannot help thinking, more accurately known, by our own writers on ophthalmic surgery.

Many facts observed at the Hôtel Dieu, have induced M. Dupuytren to admit an hereditary disposition to cataract. The following illustration tends to prove the disposition to the disease, as well as the success of the operator in removing it.

An elderly woman, accompanied by part of her family, presented herself at the hospital. She stated that, when between 60 and 70 years of age, her sight became impaired; 18 months afterwards, both her crystalline lenses were entirely opaque. M. Dupuytren depressed one, and the operation was so completely successful, that at the age of 80 she saw well. The sight of her daughter began to be impaired at 28 years of age; in a short time she could only distinguish day from night, but the pupils were moveable and the eyes healthy. At the age of 30, M. Dupuytren performed a similar operation to that to which her mother submitted, and with the same success. The daughter of this woman, aged 17, had also two cataracts, for which the Baron successfully operated; and, at the same time, the grandmother brought to the Hôtel Dieu two of her other grandchildren, in the elder of whom the crystalline lenses had begun to become opaque, whilst the younger saw objects as if through a cloud, the precursory symptom of the family disease.

M. Dupuytren has operated on a large number of congenital cataracts, but he has never seen those surprising effects of which so many authors have spoken, nor has he heard, from the lips of the patients whose sight he has restored, those marvellous reasonings on the distance, form, and colour of objects, the recital of which has produced so many metaphysical speculations. He has, on the contrary, almost always remarked, that those blind from cataract, which existed either from birth or during many years, so that they were accustomed to the exercise of but four senses, were generally embarrassed by the acquisition of the fifth; they experienced difficulty in combining the action of it with the others, and they showed frequently so great an unwillingness to employ it, that he has been obliged several times to deprive them of one, or even two of their senses, in order to force them to use that of sight. He thus stopped up the ears of a child, who guided itself either by sound, or by the impressions it received by its hands, which it carried before its body constantly, like tentacula. M. Dupuytren prefers depression of a cataract to extraction, and has rarely recourse to this operation, unless the alteration which the lens or the membrane has undergone is such as to render absorption impossible. In surgery, as in medicine, the same methods of treatment cannot be employed, in every case, to compass the same end, and no precepts are more irrational than those which recommend one operation in all cases. Relatively to age, and the degree of energy of the absorbent system, it would appear that it is better to operate on children by depression, and on old people by extraction. In children, the functions of life are carried on with all their energy, the actions of composition, and of decomposition are executed with astonishing rapidity, the crystalline is always softer than at an advanced age, and, when placed under the necessary conditions, is instantly absorbed; in the aged, on the contrary, the reverse of this is the case—absorption has lost great part of its energy, exhalation predominates, whilst the lens is harder, and absorbed with greater difficulty. M. Dupuytren has found crystalline lenses perfectly unabsorbed, although depressed for upwards of two years, in old people who had died of other diseases. But there are other conditions which favour the operation of depression in both ages; children are rarely docile, or can distinguish between that which is hurtful or advantageous to them, and their consequent resistance to an operation on the eyes renders extraction difficult, and may produce an evacuation of the vitreous humour. In old people, the eye may be deeply imbedded in the orbit, or the borders of this cavity may be very prominent, or the globe of the eye be of small size, and in such cases extraction is particularly difficult. Individuals are seen, of all ages, in whom, owing to an aberration in the motions, or in the conformation or relations of the organ, this operation is one of great difficulty; in others, the eye is agitated by rapid and convulsive motions; finally it is a constant observation, that when an individual is deprived of sight for some time, he loses, with the habit of seeing, the habit of looking, the motions of the globe no longer obey his will, and the difficulties of extraction are consequently increased. Such are some of the considerations which induce M. Dupuytren to prefer the operation of depression. He regards the operation of absorption, or that of breaking up the lens, as a modification of that of depression, and that one or the other is to be employed, according to circumstances, that whilst a hard cataract cannot be broken up, a soft one cannot be depressed. The needle which M. Dupuytren uses combines the properties of the old lance-shaped needle with the needle of Scarpa. Its blade is narrow, elongated, and bent at the extremity, its point is very acute, and both its edges sharply cutting, the size of its shaft is exactly proportioned to that of its blade, so that it is equally formed for penetrating, dividing, seizing, and displacing, readily yielding to the hand, and moving without effort, or without allowing the aqueous humour to escape. For extraction, M. Dupuytren uses Richter's knife. When the crystalline is depressed, M. Dupuytren examines carefully whether the capsule is perfectly black and detached; if some fragments still remain, he carries them into the anterior chamber. If the cataract is membranous, either partial or complete, with or without opacity of the lens, the operation is conducted in the same manner. The milky cataract requires to be broken up. If the softening is so complete, that the moment the capsule is torn a thick fluid fills the anterior chamber, so as to obscure the remaining steps of the operation, M. Dupuytren waits until absorption renders the eye again clear.

About 23 years ago, M. Dupuytren was led to introduce the needle through the cornea to depress a cataract, as his patient was a young girl whose eyes he could not fix, owing to a convulsive action of their muscles. He was not aware that this had been attempted previously; subsequently, the favourable mode in which the Germans spoke of this operation, or keratonyxis, induced him to put it to the test of experience. The consequences at which he arrived are, 1st, That it is not more easy than the operation of piercing the sclerotic. 2d, That the situation of the hand of the operator, and his needle, being between his eye and his patient's, prevents him from following the motion of his instrument or of the cataract with facility, especially when, in depressing it, the hand as well as the handle of the needle requires elevation. 3d, That the pupillary margin impedes the motion of the needle, both in depression, and in detaching the portions of capsule which adhere so often to the ciliary processes. 4th, That keratonyxis neither prevents the nervous nor inflammatory consequences which result occasionally from the posterior operation, and which have been so much dwelt on by the Germans. 5th, That iritis is a more frequent consequence, owing to the greater fatigue which the iris undergoes in this operation. 6th, That it is sometimes followed by an opaque cicatrix—in some instances, a blemish, in others, an obstacle to vision. 7th, finally, That the results of both are very similar. A table is given of the result of 21 operations by keratonyxis, 17 of which were successful; this does not differ much from the results of the posterior operation. M. Dupuytren by no means concludes that keratonyxis should be abandoned, but regards it as a new resource, preferable, in some few cases, to the ordinary method of depression. The circumstances in which it would be indicated are, prominence of the orbit, narrowness of the opening of the eyelids, smallness and depression of the globe, excessive mobility of this organ, and especially when caused by those convulsive motions to which children, affected with congenital cataract, and adults with central membranous cataract, are peculiarly liable. The following are the general principles on which M. Dupuytren conducts the treatment of his patients, before, during, and after the operation.

Previously to the operation, particular attention is paid to the state of the atmosphere and the prevailing diseases; thus, if ophthalmias are extremely common, the operation is delayed. The general health of the patient is to be carefully examined; if any rheumatic affection is present, an operation is often followed by severe inflammation; it is advisable, in the first place, to attack the rheumatism, and, if it is decided to operate when some vague pains are still present, a blister should be applied to a part at some distance from the head. If there is pulmonary catarrh, the paroxysms of coughing not only produce a greater flow of blood to the head, but they may cause the cataract to rise into its old situation. If the stomach is affected, vomiting may act in the same mechanical manner, whilst, from the sympathy which exists between the stomach and the eyes, various morbid complications may be expected. Diarrhœa and constipation contra-indicate the operation: it should not be performed during an attack of piles; and, if practised after the disease has ceased, the slightest symptoms of sanguineous congestions of the head should be combated by leeches to the anus. Even when there exists no complication, M. Dupuytren never neglects preparatory means, which are, perhaps, as important as manual dexterity. These consist in the prescription of baths, emollient lavements, and drinks, general or local bleeding, according to the circumstances, and the constitution of the patient; from time to time, small doses of castor oil, and, if the eye is very mobile, and is likely to be irritated by the approach of instruments, it is of service to habituate it beforehand to the operation, by simulating the various movements that will be required. More particularly when M. Dupuytren performs keratonyxis, he dilates the pupil, by dropping between the lids, on the preceding evening, some drops of a solution of extr. belladonnæ, or lauro-cerasus. M. Dupuytren always performs the operation whilst the patient is lying in bed, with his head elevated; this maintains the eye, as well as the whole body, in a perfect state of rest—it counteracts the tendency to syncope which sometimes occurs, and it prevents the motion of the patient from causing the cataract to remount into its former situation. He attaches considerable importance to this precaution. Subsequently to the operation, low diet and absolute repose are enjoined. If the patient is sanguineous, and there is a tendency to congestion about the head, he bleeds, &c. If there is a disposition to vomiting, which is almost always the case in children, he gives an anodyne. If there is much agitation, or nervous symptoms, lavements, with some drops of laudanum, are very useful. In those cases where both eyes are affected, M. Dupuytren never operates on more than one at the same time, the reasons of which are very obvious. It is by no means an infrequent occurrence, that the cataract remounts, and this is the most serious objection to the operation. Under such circumstances, it is better to depress it again, as, if left to itself, it is absorbed very slowly. In some subjects, there is so great a tendency to this rising of the lens, that M. Dupuytren has been obliged to repeat the operation four times at an interval of some months. Part of a cataract, or many portions of it, may rise in the same manner, requiring, in some instances, mechanical removal from the axis of vision. The lens, when diseased, appears more susceptible of displacement than before it had lost its transparence; sometimes it is wholly displaced, passing into the anterior or posterior chambers—sometimes, remaining only partially fixed, it floats about, obstructing incompletely the pupillary opening. By such displacements, patients with cataract have suddenly recovered their sight after a brisk movement, or a blow in the head or eye. Some individuals have possessed the power of making the lens pass from one chamber of the eye to the other at will. The following curious case, extracted from the work of Demours on the diseases of the eyes, proves this.

The patient, in the presence of the author and two other surgeons, caused the opaque lens to pass into the anterior chamber, and afterwards to return behind the iris. The cataract was formed at six years of age, and towards puberty, gradually descended behind the iris. At 18 years of age it was not to be seen, but was buried in the disorganized vitreous humour. At the age of 19, during an active military service, this body passed before the iris, and produced such constant pain, that the young man obtained his discharge. M. Demours was consulted and recommended extraction, but as the patient wished to avoid the operation, he advised him to dilate the pupil with belladonna, to remain lying on his back for 24 hours, and from time to time to place his head with the vertex lower than the neck, so as to favour the return of the lens behind the iris. When the lens was no longer visible, he ordered a few drops of vinegar to be dropped into the eye, to excite inflammation and consequent contraction of the pupil. This was followed with the success he expected. For 8½ years the patient was free from this singular accident; but during the last two years it has returned three or four times a month. If inadvertently he suddenly depresses his head, the lens passes before the iris; he then is in pain, and incapable of occupation, until, by lying on the ground, his chin elevated, and the summit of his head depressed, he forces it back by strongly rubbing the globe with the upper eyelid. M. Dupuytren enters at some length into the complications of cataract with adhesions of the iris, and permanent contraction of the pupil, and recommends the employment of belladonna when the effusion of lymph is recent, but when it is organized, he directs the adhesions to be broken down with the needle previously to depressing the lens.

In displacements of the crystalline lens, M. Dupuytren recommends, as a general rule, that no operation should be attempted unless the presence of the foreign body causes inflammation. The following case shows the adaptation of a new operation to peculiar circumstances, and illustrates not only the skill of the operator, but the resources of a first-rate surgeon.

A soldier, aged 34, was received into the Hôtel Dieu, the 2d Nov. 1819, having the anterior chamber of the left eye completely filled with the pearly opaque lens, which had passed through the iris whilst the patient was strongly depressing his head. The eye was red, inflamed, painful, and watering, with intense head-ache. Bleeding from the arm, a warm-bath, and a purgative, relieved these symptoms, and two days after the following operation was performed. The patient being placed in bed, with his head elevated by pillows, the operator plunged the needle into the sclerotica, about two lines from its junction with the cornea, passed it from the posterior into the anterior chamber, hooked the crystalline lens, withdrew it into the posterior chamber, and depressed it in the vitreous humour. The patient could immediately see the hand of the surgeon, and distinguish his assistants. The results were most successful, the patient left the hospital six days after with a perfectly clear pupil, seeing well, and experiencing no pain.

Such are M. Dupuytren's principal ideas on cataract. We have given them perhaps at greater length than our space will well permit, considering the value of the remaining matter, as we were the more anxious to make our readers acquainted with his views on this subject, as they differ in some points from those of our modern ophthalmic surgeons. By them extraction is generally performed, and, as our eye institutions testify, with great success; but the difficulty of the operation, and the experience required before the surgeon becomes a sure and successful operator, is allowed by all.

Pope's apothegm on different forms of government, that—

Whate'er is best administered, is best, may be applied with some restrictions to varieties in surgical operations. No one who has seen an experienced surgeon pass his knife steadily through the cornea, and extract the lens without injuring any other part of the organ, will deny that such an operation so performed, is more workman-like and physiologically correct than that of thrusting a needle through the sclerotic, choroid, and retina, and breaking up a considerable portion of the vitreous humour by forcing the opaque lens into it; or that it possesses many advantages over the slower method of passing the needle through the cornea, and subjecting the lens to the tedious process of absorption, which indeed, in many instances, is not available. But it should be recollected that this dexterity was not acquired except by such a share of practice as falls to the lot of comparatively few, and that there is some truth in the strong, but exaggerated observation of Wenzel, that a hatful of eyes must be lost before expertness is obtained in extraction. This should reconcile the provincial surgeon under whose care the poor necessarily fall, who are not unfrequently disabled by this affection, and to whom vision is almost necessary to existence, to a more simple mode of proceeding, which, although it offers many disadvantages, possesses the superiority of being more easy of execution. We are very far from agreeing with M. Dupuytren as to the peculiar advantages of the posterior over the anterior operation; we have stated his arguments, many of which, we think, anything but satisfactory, at length; but we have not now space to discuss them. To those who may wish to see the principal advantages of the anterior operation, we would recommend the perusal of an excellent and practical paper published by Dr. Jacob in the 4th volume of the Dublin Hospital Reports. M. Dupuytren has unaccountably passed over without observation the unfavourable nature of those cases which are complicated with adhesions and consequent contractions of the pupil. He gives directions for breaking them down previously to depression; but how often are they complicated with a disorganized state of the retina, and how rarely is the patient benefited by the removal of the opaque lens.

### Art. III.—On Inflammatory, Scrophulous, and Venereal Enlargements of the Testicle.

This article is devoted to the description of these three affections in order to prevent the unnecessary extirpation of the organ, a practice but too common in Paris. One surgeon indeed of great reputation is said to remove every testicle which is the seat of chronic enlargement. In this country more caution is observed, and these glands are rarely extirpated, unless the enlargement is of a well-marked malignant character, before every other means of cure have been tried. In performing the operation, M. Dupuytren makes a most extensive incision from the inguinal ring, to below the inferior extremity of the tumour. He transfixes the spermatic cord with a tenaculum previously to dividing it below that instrument; he applies two or three points of suture to the divided scrotum. Inflammation of the body of the testicle is attended with much greater enlargement, and is more easily cured, than when the epididymis is affected. The structure of the parts explains this. In such cases M. Dupuytren employs at first a vigorous antiphlogistic treatment, such as repeated venesection and leeching, baths, low diet; by these means the swelling is dissipated generally in eight or ten days. If the enlargement commences in the chronic form, or if it passes from the acute to the chronic stage, he prescribes resolutives, such as diachylon, or mercurial plasters, together with derivatives, such as purgatives, every two or three days; for this purpose he uses calomel, or castor oil, sulphate of soda, &c. The scrophulous enlargement of the testicle commences frequently in exactly the same manner as the simple inflammatory; it does not however yield to the ordinary means, and is prolonged indefinitely; it exists often with other affections of the same nature in a scrophulous habit. The tubercular degeneration affects generally the fibro-cellular structure surrounding the epididymis, as well as the substance of the gland. The tubercles increase slowly, and may remain three or four years; these are diagnostic marks. The scrophulous enlargement is less hard than the scirrhous, and harder than the inflammatory; it is without heat and redness, giving a sense of weight, and "engourdissement;" the subcutaneous cellular tissue is generally free. The tumour is commonly unequal and irregular, whilst in schirrus it is globular, and the epididymis knotty. As the disease progresses, parts of the testicle become softened; so that when touched they feel soft: these are gradually elevated and ulcerate, discharging a serous pus, and a cheesy scrophulous matter. This state may remain for years. If medicine fails, the testicle becomes soft, and fungoid, and extirpation alone can cure the disease; that is, if it is confined to this organ.

*Treatment.* The inflammatory symptoms having been subdued, constitutional remedies should be adopted The residence of the patient should be in a dry and elevated place, exposed to the South. He should be covered with flannel from head to foot, and use dry frictions; he should take exercise in the open air, and as much as possible in the sun; his diet should be strengthening, consisting of brown meats, game, and antiscorbutic vegetables, avoiding mineral and vegetable acids, sour and especially farinaceous vegetables. Iodine may be employed in doses of an eighth to half a grain in mint water, or frictions with hydriodate of potash. Salt, sulphur, or ioduretted douche baths may be used, particularly when there are fistulous sores. If, notwithstanding these remedies, the disease proceeds, and the testicle becomes soft, fungoid, and full of scrophulous deposits, or if it threatens to pass into scirrhus, which is rare, extirpation should be employed without hesitation, or the patient is exposed to some fatal organic disease.

Patients constantly present themselves with enlargement of the testicle, for which they can assign no cause; they have received no blow, nor other external injury. If the tumour is elongated and cylindrical, if it causes no lancinating pains when touched, if the patient has had former venereal affections, if he states that. after being six, twelve, or eighteen months affected, the testicle has returned to its normal state, and the other has enlarged, there are strong presumptions in favour of the venereal nature of the disease; the enlargement shifting from one testicle to the other is a pathognomonic symptom. In cases where the disease returns after castration, if a cancerous testicle has been removed, the cord almost always becomes affected; but, in syphilitic enlargement, the remaining testicle is the seat of the affection. When there are other syphilitic symptoms, such as eruptions, nodes, &c. the diagnosis is more clear. If there is doubt, it is better to adopt a syphilitic treatment during six weeks or two months, than risk an operation. M. Dupuytren's antisyphilitic treatment consists of the compound decoction of sarsaparilla and the following pills—

℞. Hydrargyri oxymur .... gr. $\frac{1}{8}$ to $\frac{1}{2}$
Opii . . ............ .. gr. ss.
Extr. Guaici ... ...... gr. ij. M. Fiat pilula, ter quotidie sumenda.

### ART. IV.—TRAUMATIC EMPHYSEMA.

Two cases of emphysema, from fracture of the ribs, are detailed; one of these was mild—the other severe and fatal; they very fairly illustrate the subject, and introduce some useful remarks, but, as there is nothing novel in the article, we shall pass on to a case of traumatic emphysema of the eyelids.

A workman, aged 25, whilst excavating, had the right anterior part of his head, his neck, and chest covered by the falling-in of the earth. On being extricated, he merely felt slight pain at the root of the nose, to which he paid little attention, and continued his work. About a quarter of an hour afterwards, having endeavoured to blow his nose, the eyelids became suddenly and considerably tumefied, so that the eye was entirely covered. On touching the part, there was evident crepitation. M. Dupuytren considered that the external violence had ruptured the pars plana of the ethmoid bone, or the os unguis, and that, through this opening, the air passed from the nasal fossæ into the internal structure of the eyelids. The reason why the emphysema did not appear immediately after the accident, but not until the man had endeavoured to blow his nose, was, probably, that the fracture of the bone was unattended, at first, with destruction of the soft parts which line it, and which formed an obstacle to the transmission of air; but that subsequently, the patient having, by his efforts to blow his nose, driven a strong current of air against these parts, they had given way, and established a communication between the nasal fossæ and the eyelids. Bleeding from the nose is a usual symptom in such cases, but in this it did not occur. Venesection was employed, and wet compresses were applied to the base of the orbit. M. Dupuytren recommended the patient to avoid blowing his nose, coughing, or any other effort which could renew the passage of the air through the presumed opening. On the third day the crepitation had diminished, and, on the fifth, the eyelids were in a natural state.

*Emphysema of the Temporal Region.* A man fell on the anterior part of the forehead; some time after a large tumour formed in the temporal region, the nature of which appeared doubtful to several persons. M. Dupuytren examined, and, by slightly compressing it, caused it to move towards the anterior part of the forehead, and, finally, to disappear. It was owing to the passage of the air from a fracture of the frontal sinus into the cellular tissue.

In the diagnosis of cases of this kind, besides the emphysematous crepitation, it is observed that the swelling is increasd when the patient blows his nose; and, if he is in a state of insensibility, the same effect is produced by pinching his nose, so as to prevent the expired air from passing through it. The swelling always forms with extreme rapidity, owing to the laxity of the cellular tissue.

### Art. V.—On Fistulous Sinuses and Symptomatic Abscesses.

A woman was admitted into the Hôtel Dieu on account of an abscess on the inner and upper part of the thigh. There was considerable curvature of the spine, owing to caries of the bodies of some of the vertebræ. A smaller tumour existed on the other thigh. It was clear that these abscesses communicated, by two fistulous sinuses, with the diseased bone. Moxas were applied to the sides of the spine corresponding with the diseased vertebræ, and in a month the symptoms were ameliorated, when she was attacked, owing to a change of temperature, or perhaps to an absorption of pus, with pleuro-pneumonia, which proved fatal.

On examining the body, 36 hours after death, the following appearances were observed, which we shall transcribe, as they illustrate M. Dupuytren's remarks on the formation of these fistulæ. The body of the 11th dorsal vertebra was destroyed by caries; the vertebral canal was not contracted, neither had the spinal cord lost its natural appearance. The bodies of the 10th and 12th dorsal vertebræ were superficially carious and softened. In front of the 11th dorsal vertebra, the condensed and hypertrophied cellular tissue and periosteum formed a pouch, whose walls were thick and resisting, having a grey internal surface, in contact with pus and purulent pseudo-membranes; it adhered by strong bands to the bodies of the diseased vertebræ. From this pouch, two fistulæ passed downwards within the sheath of the psoas muscles, which were pale and atrophied. The right was the larger, and would admit many fingers; it contained unhealthy serous pus—it was lined by thick pseudo-membranes, beneath which was a smooth, rosy, and apparently mucous membrane. It was dilated above the crural arch, contracted under it, and again enlarged, at the upper part of the thigh, into a large sac, containing the small trochanter, with new bony depositions; this communicated with the fistular opening of the skin. The left fistular tract followed a similar course, was lined by a recent mucous membrane, but was not dilated; beneath this membrane, a whitish, resisting, fibrous tissue, formed almost the whole of the canal, and was evidently produced by the cellular tissue of the parts through which the pus made its way. This sinus was evidently undergoing a cure, as it was in some parts capillary.

After bringing forward this case, M. Dupuytren remarked, that those channels which establish a communication between a carious bone and any other part of the body, those which pass from the urethra to the perinæum, from Stenon's duct to any part of the face, or between the air-passages and the external surface, all present the same nature and organization. They are formed at the expense of the cellular tissue of the parts through which the pus passes. The fibrous, nervous, osseous, or mucous tissues may thus furnish cellular membrane, in which the granulations appear and become united together; their canals, constantly traversed by their products, speedily assume a mucous character. In caries of the spine, the fistulous sinuses are thus formed. Caries commencing, the pus remains for a longer or a shorter time in the diseased bone, or in the parts which surround it, particularly in the cellular tissue; a cyst is there formed, where the matter accumulates. The quantity of pus increasing, and gravitating, the cyst becomes elongated, and directed either to one or both sides of the spine; the pus then advances, by driving before it the inferior extremity of the cyst; if it meets with an obstacle, it forms a dilatation; it contracts when pressed between the parts it may pass through, and again dilates when no such impediment exists. Having arrived at the skin, it forms a sinus beneath it of greater or less length, and a tumour is produced, which becomes an abscess. This is the congestive abscess of authors (but M. Dupuytren prefers the term symptomatic), and constitutes a dangerous and generally fatal disease. By active counter-irritation and constitutional remedies, the caries may be often arrested and cured, but the abscess may remain many years without any injury; in some cases it is gradually absorbed; in others, after a certain time, the skin inflames and ulcerates, and the pus is discharged without being again renewed. In some circumstances, the pus has been converted into a substance which chemistry has proved to be adipocire, as in the following case.

Many years ago, M. Dupuytren attended a young merchant, who was affected with symptomatic abscess, arising from caries of the spine, attended with considerable curvature; the caries yielded to the repeated application of moxas, cauteries, &c.; the abscess did not disappear, but its size was slightly diminished. Five or six years afterwards, the patient died of a pleuro-pneumonia. On examination, the caries was found cured, the deformity alone remained; the abscess was converted into a fatty, soft, unctuous matter, presenting all the physical and chemical characters of adipocire, the sinus extending from the diseased bone to the abscess was contracted, and, in some parts, obstructed; its walls still contained this peculiar matter.

M. Dupuytren considers it dangerous to open symptomatic abscesses, from caries of the spine which has yielded to the curative means, as it exposes the patient to a return of the disease. He leaves these abscesses, under all circumstances, to the efforts of Nature. The analogy in structure between these artificial canals, and natural mucous canals, was exemplified in the examination of the first case. There was a false membrane, analogous to that in inflammation of the œsophagus; on scraping this off, the subjacent membrane was red, like natural mucous membranes—it was, like them, soft and villous, the villi being easily seen with a lens, less, certainly, than in a natural state, but still well marked. On the exterior was a fibro-cellular membrane, similar to that which surrounds mucous membranes. In some instances, Nature cures these artificial sinuses; the mucous canal, ceasing to be traversed by a fluid, contracts, its sides approach each other, unite, and form a fibro-cellular cord, which, in the course of six or twelve months, wholly or partially disappears. This has been fully proved by pathological examinations. The reason that the natural mucous canals are obliterated with such difficulty and so rarely, whilst the contrary is the case with the artificial, appears to arise from the abundant apparatus for secretion with which the former are furnished; for, as in a square inch, the natural canals have 100 villi—the artificial have not above 5 or 6. From these considerations, the necessity will be seen to oppose, as soon as possible, the organization of these fistulæ, and to hasten to re-establish the course of the secretion by all suitable remedies. But when this object cannot be obtained, such means become unsuitable, and the parts themselves must be removed. If the canal is straight, and the actual cautery can be applied to its whole internal surface, it may be useful. In other cases, injections of nitrate of silver, or nitric acid in a large quantity of water, may be carefully thrown in. M. Dupuytren employs a solution of 30 grains, or a drachm, of nitrate of silver in a pint of distilled water.

### Art. VI.—Prolapsus of the Rectum.

Prolapsus of the rectum is one, if not of the most serious, at least one of the most inconvenient diseases; in general, it occurs in those who are the subjects of it whenever they go to stool, or even when they remain standing, and it appears to co-exist with a weak, soft, lymphatic, and hæmorrhoidal constitution. Reduction is only a palliative remedy: cold baths have been recommended as curative means, but they require much time and perseverance, so that they rarely fulfil the intention. Astringents, compression with sponge and a T bandage, suppositories, &c. have been of use in the prolapsus ani of children, but have failed in adults and the aged. Excision of a portion of the mucous membrane, cauterization, &c. are not only excessively painful, but dangerous. The inefficacy of these means has induced M. Dupuytren to practise a new mode of treatment, and, previously to explaining it, a short anatomical description of the parts will not be out of place.—The skin surrounding the margin of the anus is thinner, and differs in colour from that covering other parts of the body; it contains numerous mucous crypts, which secrete an oily matter. This skin is thrown into folds, separated by furrows, which converge from the margin towards the centre of the anus; these folds are implicated in the anus itself, and are more numerous and prominent in proportion as it is closely contracted; they disappear when it is dilated, and it is readily conceived that their use is to facilitate the dilatation of the anus, and to favour the expulsion of the fæces. Above the skin is a layer of fibro-cellular substance—above that the external sphincter, and above that the internal sphincter.

The operation which M. Dupuytren has proposed is thus performed:—The patient lies on the belly, the upper part of the trunk and the head low, the pelvis, on the contrary, much elevated by pillows; the thighs and buttocks are separated, to expose fully the margin of the anus and the anus itself. The operator, holding in his left hand a pair of dissecting forceps, with large blades, in order to give less pain, seizes successively, from right to left, and even from before and behind, two, three, four, five, or six of these diverging folds; with a pair of curved scissors, held flat with the right hand, he cuts off each fold as soon as it is raised: the excision should be prolonged as far as, and even within, the anus, as the action extends even above the opening; if the relaxation is considerable the incision may be carried to half an inch in length, but in general a few lines is sufficient. If the relaxation is moderate, it is sufficient to remove two or three folds on each side; if it is very great, a greater number must be cut off. This operation gives but little pain, and no inconvenience. There is no hæmorrhage, as the cutaneous vessels alone pour out blood. The cicatrization which causes at the same time the edges of the wounds to approach each other, and the formation of an accidental tissue, evidently contracts the anus. A new skin intimately adhering to the parts beneath has been substituted for the former skin, whose adhesions were remarkably lax. The inflammation which follows, and is propagated to the submucous cellular tissue of the rectum, tends to unite the mucous and muscular coats more intimately. No dressing is necessary. During the first days after the operation the patients do not ordinarily go to stool, but the inflammation soon subsides, the cicatrization is complete in a few days, the opening is diminished, and the cure perfect. It is ten years since M. Dupuytren first performed this operation, and since that time he has employed it in a great number of cases, and always with the same success;—the disease has never returned, unless from the indocility of a child rendering the operation imperfect.

*Case.* A young woman, of good constitution, subject to prolapsus ani, entered the Hôtel Dieu in May, 1830. She knew no cause to which the disease might be attributed. Whilst in the hospital she was not subject to piles, but there was this peculiarity about the prolapsus of the rectum, that it was only troublesome during some days in each month, whenever the patient went to stool. The extreme pain she suffered whilst the parts were protruded made her wish for an operation. The patient being properly placed, M. Dupuytren raised with his dissecting forceps a fold of the margin of the anus, and removed it, prolonging the incision as high as possible into the rectum. Four folds were thus removed from before, behind, and the two sides. The pain was but moderate, and there was no hæmorrhage; no dressing was applied, and on the patient's going to stool some days after, the gut did not descend. In a fortnight the wounds were healed, and the patient was discharged. A very similar case is given in which three folds were removed; the patient was a child three years of age.

Although he, and subsequent writers, have given cases in which prolapsus ani of the rectum was cured by removal of a portion of the skin around the anus, yet these cases have been complicated with hæmorrhoids, for which alone the operation was attempted. To M. Dupuytren must be given the full credit of this ingenious operation, which still farther differs from those which have been previously attempted, from its being peculiarly adapted to those cases which are *not* complicated with piles.

### ART. VII.—ON NERVOUS OR TRAUMATIC DELIRIUM.

The details of seven cases of what M. Dupuytren calls nervous delirium, and which has usually been named "Traumatic" from its cause, are given. In three of these the delirium followed fractures of bones, in two it was subsequent to self-inflicted wounds, in one case it followed the removal of a sarcocele, and in the last it was preceded by an operation for the removal of cataract. Only one of these terminated fatally. At the conclusion of the article M. Dupuytren has summed up the distinguishing symptoms. It commences generally in a sudden and unexpected manner in individuals often in a very favourable condition, by gestures, disordered movements, incoherent questions, and a singular confusion of ideas respecting places, persons, and things. Completely deprived of rest, they appear under the influence of one idea, more or less fixed, but almost always connected with their profession, their passions, their tastes, their age or sex. They are in constant motion. The upper parts of the body are covered with an abundant perspiration; the eyes become brilliant and injected, the face animated and red, and they utter menacing words and frightful vociferations with extraordinary loquacity. Their insensibility to pain is often so great that patients with comminuted fractures of the lower extremities have been known to bear off the dressings, and walk about on the maimed limbs without any expression of suffering; others, with fractured ribs, have agitated themselves and sung without the slightest uneasiness; and some, after being operated on for hernia, have introduced their fingers into the wound, and amused themselves by drawing out their intestines as coolly as if they were performing the manœuvre on a dead body. Notwithstanding the severity of these symptoms, the pulse is tranquil and calm, experiencing no other change than that produced by the disordered movements of the body; there is no fever, the excrementitious functions are executed with their usual regularity, but the appetite is gone, and at the end of two, four, or five days, the disease terminates by death, or more frequently favourably. The favourable change takes place suddenly, and without an apparent crisis. Exhausted with fatigue, the patient falls into a profound and tranquil sleep, awaking in ten or fifteen hours with restored reason, without remembrance of the past, but weak and sensible to pain; the appetite returns, and the primary disease pursues its usual course. The delirium may re-appear two or three times after one or more days of remission, but at each return it is more feeble. The most striking symptom is the calm state of the circulation and absence of all febrile symptoms, notwithstanding the disturbed state of the sensorial functions; the patient is furious, out of his mind, his face inundated with perspiration, his eyes sparkling, his cries loud, but his pulse is calm and regular, and the state of skin removes every suspicion of inflammation. It is a true mania, but of short duration. Those who are nervous and pusillanimous, or whose head has been overset by a strong and quickly-conceived resolution, are most liable to this delirium. Thus it is very common among suicides; but athletic men are not exempt. Women are less exposed to it, and it has not been observed among children. M. Dupuytren, in the great majority of cases, founds his prognosis on the accompanying disease. Thus, if the delirium follows a fracture of the bones of the limbs or chest, the prognosis will be more unfavourable than if it succeeded simple wounds which were not in themselves dangerous. Post-mortem examinations do not explain the symptoms. As narcotics taken into the stomach, as well as depletion, &c. have generally failed, M. Dupuytren has been led to adopt a mode of administering sedatives, which has so constantly succeeded that he is inclined to call it a specific. It consists in the administration of some drops of laudanum in a small glyster. Five or six drops of laudanum in a four-ounce lavement produce more effect than three times the quantity taken into the stomach. These should be repeated three or four times at intervals of six hours. This superiority is owing to the surface of the rectum being a purely absorbing surface, whilst medicines given by the mouth are subject to the action of the gastric juices; to this must be attributed the uncertain effect of so many vegetable narcotics.

### Art. VIII.—Fractures of the Inferior Extremity of the Fibula, and Luxations of the Foot.

Although the fibula is weaker than the tibia, and more exposed to external injuries, it is less frequently fractured. This is to be attributed to its not transmitting the weight of the body to the foot, to its elasticity even in old age, and to the protection from external violence which it receives from the numerous muscles which surround it. But fractures of the fibula have been reported by writers to be of less frequent occurrence than they really are, owing to their being frequently mistaken, and often confounded with luxations of the tibio-tarsal articulation. M. Dupuytren estimates that fractures alone of the inferior extremity of the fibula are to the rest of fractures of the leg as one to three. The powers which produce fractures of the fibula act either directly upon it, or indirectly through the medium of the foot; hence two kinds of fractures, differing in their causes, effects, and curative indications. Direct violence produces fractures of the middle and upper part of the bone, and are less frequent than its situation and form would indicate; owing to the rarity of such causes, and the protection which the bone receives from the peronei muscles. These fractures are remarkably analogous to those of the ulna, which M. Dupuytren asserts is never broken by itself, unless by violence immediately applied. If the tibia remains unbroken, there is no displacement as to length; the foot preserves its normal direction, and the depression caused by the fractured ends of the bone being slightly driven inwards, is felt with difficulty. On this depression, as well as the circumstances connected with the injury, as the violence of the blow, the weight of the body which has struck the limb, the existence of a large ecchymosis and violent contusion, and the ease with which the fractured ends are depressed towards the tibia, must the diagnosis be founded. Sensible crepitation can rarely be produced, owing to the small size of the broken ends, and the exactness of the relations which they preserve. In simple fractures it is merely necessary to keep the limb immoveable by the usual apparatus. The fracture is consolidated in thirty or thirty-five days. Fractures of the malleolar extremity of the bone are much less simple; their results are frequently unfortunate, and they have been constantly confounded with luxations of the foot. Notwithstanding they had attracted the particular notice of Petit, Pott, Boyer, and many other celebrated surgeons of both countries, M. Dupuytren, who has paid great attention to the subject, has rendered the history of these fractures more complete, and their management more certainly efficacious.

*Causes.* The inferior extremity of the fibula may be broken by direct violence, or indirectly through the medium of the foot. Accidents of the first class present no remarkable characters, whilst those of the second demand the closest attention of the surgeon, especially as to its causes, that he may be aware, in doubtful cases, of the possibility of the existence of this injury. A stone, an excavation, or even a simple inequality of the ground, a fall from a place more or less elevated, on the feet, bent either inwards or outwards, are the most common causes of these accidents; they are the immediate result of the weight of the body and of musular contraction acting suddenly and violently on the inferior articulation of the leg, at the same instant as the foot carried either inwards or outwards is removed from the vertical line. If the foot is forced inwards, the line of the transmission of the weight of the body, instead of intersecting the axis of the tibia, and falling on the astragalus, passes obliquely from within outwards, through the inferior extremity of the tibia, the articulation, the external malleolus, and the outer side of the foot. The weight of the body is then supported by the outer ankle, and the inferior extremity of the tibia; and the external malleolus, or even the inferior extremity of the fibula, give way, from the traction of the external lateral ligaments, which exert more force from their being placed in a direction almost perpendicular to the malleolus, when it is resting on the sharp edge of the astragalus, which is forcibly driven from within outwards by the tibia. This last bone, thicker and stronger than the fibula, in general resists, and if its malleolus or inferior extremity are broken, it is not primitively, and by treading with it again, but consecutively, and by the displacement of the foot outwardly. When the foot is forced outwards the centre of gravity of the leg traverses obliquely the inferior part of the fibula, the articulation of the foot, the internal malleolus and its ligaments, and falls on the ground at a greater or less distance from the internal edge of the foot. The internal ligaments, and the malleolus to which they are attached, on the one part, and the inferior extremity of the fibula, on the other part, have to support the weight of the body and the muscular action, these are consequently torn or fractured, the lateral ligaments, or the inner ankle give way in the first place, and the inferior extremity of the fibula in the last.

*Signs.* These are of two sorts, presumptive and characteristic. The presumptive are the kind of accident, a cracking noise heard at the same instant, fixed pain in the lower extremity of the fibula, difficulty or impossibility of walking, swelling around the joint, and particularly around the lower ankle. The characteristic marks, are, an unnatural mobility of any point of the lower extremity of the fibula, crepitation on motion, the ease in which the fibula can be pressed against the tibia, the mobility of the foot in a transverse direction, the change in the point of incidence of the axis of the leg on the foot, the displacement of the foot inwards, outwards, or backwards, rotation on its axis from within outwards, an angular depression at the external and inferior part of the leg, prominence of the inner ankle, the disappearance of almost all these marks as soon as the reduction of the foot is attempted, and their immediate return when such efforts are suspended, and especially when the limb is placed in an extended position. Such is a general enumeration of the symptoms, some of which require a more detailed examination. The fibula being broken, the external side of the mortice which receives the astragalus loses its solidity, no longer resists the action of those muscles which tend to turn the foot outwards, and which now overcome their opponents. The external border of the foot is consequently raised, the internal depressed, its dorsal surface is turned directly upwards, and its palmar surface outwards, the pulley of the astragalus is directed to the inner malleolus, and sometimes forms an eminence easily distinguished beneath the integuments; the outer malleolus undergoes a kind of semi-rotation on the tibia by which its summit is elevated and it brings nearer to the axis of the limb the superior extremity of the fragment that it terminates. The foot is thus displaced from the centre of the inter-malleolar surface; so that in drawing a line through the axis of the tibia it would fall on the internal side of the tarsus, and the weight of the body would be supported by the internal angle and its ligaments. This displacement of the foot outwards is the only one which would necessarily result from the fracture of the fibula, it is more marked in proportion as the bone has been fractured near its extremity, and the patient has endeavoured to make use of the maimed limb. In those cases where the fracture results from the violent inclination of the foot inwards, the muscles speedily draw it outwards. In cases where the accident has been misunderstood, or imperfectly treated, the soft parts are distended, inflamed, and disorganized; the synovial capsule is opened, and the articular extremities become carious and are destroyed. In the most favourable circumstances the patients are never able to use the limb without the aid of crutches or a wooden leg. M. Dupuytren has collected a large number of cases proving the unfortunate effects which result from these accidents not being recognized. In passing the finger over the inferior portion of the fibula an unusual mobility, which is different from the elastic flexibility of the bone, is discovered at the situation of the fracture. This is rendered more manifest by embracing the tibia with four fingers of each hand, whilst the two thumbs are pressed alternately against the two ends of the fracture. There is generally very little crepitation, often none. In seizing with one hand the inferior part of the leg, and with the other the tarsus, the foot can, if the fibula is fractured, be alternately carried outwards and inwards. If left to itself the foot is carried outwards, the internal malleolus forms a considerable eminence, over which the skin is tensely extended, the axis of the limb falls on the inner side of the tarsus, the space which separates the two malleoli is increased, on the external side of the articulation the skin is thrown into transverse furrows, the external malleolus appears sunk, above it and at the seat of the fracture is seen almost always a sudden depression directed from before backwards, or as M. Dupuytren expresses it, "une sorte de *coup de hache*," which is a pathognomic symptom of this accident. In some cases the foot has been so forcibly bent inwards that it remains in the same situation notwithstanding the fracture of the fibula; but then the superior extremity of the inferior fragment projects against and threatens to tear the skin, and the inequalities of the fracture are felt by the finger; after the reduction of the luxation the muscles draw the foot outwards, so that this form of the accident cannot be mistaken.

M. Dupuytren divides these fractures into simple and complicated. The fracture is simple when the injury consists solely of solution of continuity of the bone. This is extremely rare and difficult to recognize; there are two varieties of simple fracture; in the first the bone is broken at upwards of three inches from the malleolus, and similar observations apply to this fracture as to those higher up, and which have been previously detailed; the second variety includes all those cases where the fibula has been broken at less than three inches above the malleolus without displacement of the foot. It most usually occurs at about two and a half inches above the extremity of the malleolus when the injury has been produced by the foot having been violently twisted outwards, because at that point the fibula is most weak and thin, and it is bent inwards by the weight of the body, and the action of the muscles. If the foot has been forcibly turned inwards, the fibula generally gives way below this, and at that part which is lodged in the groove of the tibia. Complicated fractures of the fibula are numerous. 1st. The force which bending the foot outwards and fracturing the fibula may be so violent as to rupture the internal lateral ligaments, and fracture the inner malleolus, or the same injuries may be produced by the patient's endeavouring to walk after the foot has been thus forcibly twisted inwards. 2nd. Instead of a fracture of the inner malleolus and rupture of the lateral ligaments, the inferior extremity of the tibia may be broken. 3rd. This complication, which is almost universal, is luxation of the foot. This may take place inwards, backwards, outwards, and outwards and upwards. The dislocation inwards is so commonly united with fractured fibula, that one is rarely seen without the other; it consists in a displacement of the head of the astragalus, which is placed beneath and to the inner side of the tibial malleolus, the effect either of the prolonged action of the causes which have produced the injury, or of the action of the abductor muscles of the foot. The dislocation backwards is owing to the gastrocnemius and soleus by acting on the foot, which is no longer retained by the resistance of the external malleolus, drawing the astragalus from before to behind the inferior extremity of the tibia, whilst the lower end of the inferior fragment of the fibula is carried backwards, and its other extremity forwards. The dislocation outwards is the rarest and most difficult to explain. The astragalus is then carried to the side, and beneath the external ankle, whilst the outer border of the foot is placed inferiorly, the sole inwardly, and its internal border superiorly, the tibial malleolus is hidden, and disappears at the bottom of the angle formed by the foot and the leg, and the fibular malleolus forms with the astragalus an angle, prominent, and rounded posteriorly. The foot has the appearance of a congenital club-foot.

M. Dupuytren attributes this rare accident, in which the astragalus is carried outwards and the foot inwards, notwithstanding the superior muscular power of the abductors over the adductors, to the obliquity of the fracture of the tibia, influencing the direction of the displacement, and to the resistance that the broken fragment of the bone makes to the foot being carried outwards, thus favouring the action of the abductors. The luxation of the foot outwards and upwards, and which has not before been described by authors, has been observed but once by M. Dupuytren during a practice of upwards of twenty-five years, during which time he has treated more than 200 fractures of the fibula, but the single case was so well marked as to prevent the possibility of mistaking such an accident in the future. The astragalus, the external malleolus and the foot were carried at first to the external side of the leg, and were subsequently drawn up along the tibia to the height of two inches without any separation of the different parts. Such an accident could not occur without fracture of the fibula and complete laceration of the tibio-perineal ligaments. Either one or both bones may be broken in many places and the spiculæ driven into the surrounding soft parts, the articulations and the tendinous sheaths may be opened, and the tendons, nerves, blood-vessels, &c. displaced, compressed, torn or ruptured. The blood-vessels alone may be ruptured without this formidable complication of injuries just enumerated, and the blood may be effused in the cellular tissue around the joint in large quantities. Very commonly the skin is destroyed in one or more places, either primitively by the fractured ends of the bones, or consecutively by the effects of inflammation, &c. These lesions of the skin are so dangerous a complication, that fractures, by no means serious in other respects, may produce the most dangerous consequences, whilst the cure of internal injuries, to whatever degree they may have been carried, need never be despaired of, as long as the skin is sound.

When the tumefaction and tension which result from the flow of fluids towards the injured parts are not dissipated at first, they increase in a few hours to a most dangerous degree, the limb becomes livid, cold, and insensible, phlyctenæ full of reddish serosity are formed here and there, and gangrene comes on without any previous symptoms of inflammation. But in other cases inflammation succeeds the tumefaction, and this follows two different courses; in one, the inflammatory symptoms progressively increase, until they terminate in gangrene; in the other, slow phlegmonous erysipelas produces extensive suppuration, the subcutaneous cellular tissue sloughs, the skin gives way, slow fever comes on with loss of strength, until the patient, debilitated by fever, suppuration, and diarrhœa, dies. Tetanus, and nervous delirium are often fatal combinations. The bone, or fragments of bone which by the accident have been exposed to the air, are liable to necrosis; but this is a less common sequence than sloughing of the tendons of the inferior extremity of the leg. The symptoms do not manifest themselves immediately, but after a greater or less period of time, heat, redness, pain, tumefaction, tension, and obscure fluctuation are observed along the course of the tendons, the skin is gradually thinned and destroyed, and pus, followed by the dead shreds of the tendons, escapes.

The loose application of the terms outwards and inwards to dislocations and displacements of the foot, by surgical writers, is often the source of confusion. M. Dupuytren throughout this article, when speaking of luxations, applies them to the relative position of the astragalus and tibia, but when describing displacements of the foot, to the direction in which it points, thus, in luxation of the foot inwards, there is displacement of the foot outwards, and vice versâ.

*Treatment.* Pott has described the manner in which this accident can be reduced, with facility, and without effort; but he has not pointed out any means by which the reduction may be prolonged. M. Dupuytren in his treatment of these injuries has attended particularly to this most necessary part of the curative indications, the neglect or ignorance of which has been the cause of complete deformity resulting so often from this injury.

When there is simple solution of continuity of the fibula, the sole indication is to prevent all displacement of the fragments; repose and absolute immobility attain this object. These, together with reduction are sufficient when the accident is attended with simple displacement of the foot. But it is a question of considerable importance, whether there is any kind of complication of fracture of the fibula which contra-indicates reduction? As all the unfortunate symptoms which follow, are the immediate effect of the powers which have produced the accident or the consecutive result of the fracture itself, and as they must become more dangerous the longer the cause persists, M. Dupuytren lays it down as a general rule, that the surest and quickest way to relieve these symptoms is to reduce the parts at every period of the disease. This was also the opinion of Dessault.

*Reduction.* Nothing is more easy to reduce than fracture of the fibula, attended with displacement of the foot, when the resistance of the opposing muscles is overcome. This end is obtained by bending the leg on the thigh, and diverting the attention of the patient. The resistance of the muscles is by this simple proceeding immediately overcome, and almost without effort the parts resume their natural position. Notwithstanding the apparently perfect reduction, it is always incomplete, for the fragments of bone remain depressed towards the side of the tibia, and the foot preserves a constant tendency to yield to the action of the lateral peronei muscles, and to be carried outwardly. Some method is therefore necessary to separate these fragments from the tibia, and to keep them in apposition. The superior portion is never depressed, and it is impossible to act on the inferior, except through the medium of the foot, which is closely connected with both malleoli. It will therefore be evident, that the broken external malleolus will be raised by carrying inwardly with some force the internal border of the foot, that is, placing it in the position of abduction. In this manner, the inferior extremity of the tibia is sunk deeply in the articulation, the astragalus is carried from within outwards, and the inferior fragment of the fibula performs on itself a movement of semi-rotation in a contrary direction to that which displaced it, and is thus placed in apposition with the upper fragment.

*Means employed in maintaining the parts reduced.* It is evident that the limb must be kept in the position which was necessary for its reduction. The apparatus which M. Dupuytren has employed for 25 years in fractures of the fibula complicated with luxation inwards, simply consists of a pad, a splint, and two bandages. The pad is made of linen, and filled two thirds with the chaff of oats; it should be 2½ feet in length; 4 or 5 inches in breadth; and 3 or 4 in thickness. The splint should be from 18 to 20 inches in length; 2½ inches in breadth; and 3 or 4 lines in thickness, of firm and but slightly flexible wood. The two bandages, made of partly worn linen, should be 4 or 5 ells in length. The pad, doubled like a wedge, is applied to the inner side of the fractured limb, extended upon the tibia, its base resting on the internal malleolus without passing it, and its apex on the inner condyle of the tibia. The splint applied along the pad should pass five or six inches below it, and three or four inches below the inner border of the foot. The splint and pad are then fixed to the superior part of the leg, by some turns of the bandage passed from above downwards. In this state a space equal to the thickness of the pad, that is from 4 to 5 inches, is left between the splint and the foot; this extremity of the splint serves as a point d'appui to bring the foot from without inwards. For this purpose the end of a second roller is fixed to the splint, and is applied successively over the superior surface of the foot, over its external border, under the sole, over the splint, then upon the instep, and under the heel, to return again over the splint, and to be continued in the same manner until the whole length of the roller is used. By this means, the foot is placed in such a state of adduction, that its external border becomes inferior, its sole is directed inwards, and its internal border upwards. The tibia, pressed by the base of the wedge formed by the cushion, is forced outwards, as well as the astragalus; the inferior fragment of the fibula, pressed outwards superiorly by the tibia, and drawn inwards inferiorly by its external lateral ligaments, performs that semi-rotation by which it is brought to its natural situation. In order to obtain a complete reduction, it is not sufficient to bring the foot into the same line as the leg, but it should be brought by the bandage as far inwards, as it was displaced outwards by the accident. This apparatus, besides the advantages it possesses of reducing the injury without effort, and almost without pain, and of maintaining the parts reduced, also leaves a considerable interval between the bandages, which exposes the articulation and the situation of the fracture, so as to allow the application of topical remedies, &c. The same apparatus is applicable in all cases of fracture, with simple luxation of the foot outwards.

In luxation outwards and upwards, the splint, &c. must be placed along the fibula, instead of the tibia. But the luxation backwards presents both greater difficulties in reduction, and in maintaining the parts in sitû. The difficulty of reduction depends on muscular resistance—that of keeping the parts in sitû, when reduced, arises from the superior surface of the astragalus, convex from behind forwards, being so slippery that the tibia rests perpendicularly upon it with great difficulty, being constantly disposed to incline forwards, whilst the astragalus itself, incessantly acted upon by the extensor muscles of the foot, which are more powerful than the flexors, has a continual tendency to slip behind the inferior extremity of the tibia.

In this accident, M. Dupuytren employs, besides the former apparatus, a small cushion, some inches square, filled with hair or oat-chaff. The large pad, folded as a wedge, is placed on the posterior part of the leg, extending from the heel to the hollow of the ham, its base inferiorly and its apex superiorly. The splint is applied on this pad, and is fixed to the upper part of the leg by one roller; a second bandage embraces the lower extremity of the splint and the leg, and is the really acting part of the apparatus. The small pad is placed on the tibia, to preserve it from the compression of the turns of the bandage, which, bearing on the splint and tibia, carry at the same time the heel forwards and the tibia backwards. Such is the power of this method, that the only fear is that it will be too great. In those complicated cases, where the foot is luxated inwards and backwards, the treatment must be adapted to the displacement which is most predominant. In a contrary case, is is easy to combine both methods, so as to fulfil the double indication. Seven cases are detailed at some length, supporting the principles laid down by M. Dupuytren, and contrasting the former treatment of these accidents, with his improved method. The following is a short abstract of the last case, exemplifying the unfortunate consequences which result from the delay of reduction.

A servant of M. T. fell from a tree on the inner border of the right foot; this was followed with acute pain in the joint, and great tumefaction. A country surgeon pronounced it a sprain; a second discovered, on the fifth day, the nature of the accident, but recommended leeches, emollient applications, &c. and delay of the reduction and fracture, until the inflammatory symptoms had subsided ; but they increased, an abundant suppuration of the parts took place around the joint, and gangrene threatened. M. Dupuytren was called in ; he found all the appearances strongly marked, of luxation of the foot inwards, and fracture of the fibula, and being convinced that the disorganization of the soft parts was owing to the displacement, and would continue unless the cause was removed, he proposed immediate reduction. The other surgeon opposed this proceeding, stating it to be useless, as it could be performed at a later stage without difficulty, and dangerous, from the parts not being in a fit state to support the efforts of reduction. The suppuration of the cellular tissue and sloughing of the skin continued. The symptoms were somewhat abated at the end of three weeks, and reduction was then attempted, but in vain ; during fifteen days, at various intervals, the attempt was repeated, but with no better success. Notwithstanding the subsequent erysipelas, excessive suppuration, bilious fever, &c. the patient recovered, but with permanent deformity.

The effects of the treatment recommended by M. Dupuytren are, 1, The return of the foot to its natural situation; 2, The exact reduction of the fragments, so that, when the cure is completed, no trace of the injury can be detected; 3, The instantaneous cessation of the painful suffering produced by the displacement; 4, The rapid diminution of the tumefaction, &c.; 5, The removal of all the causes capable of producing unfavourable consecutive symptoms, and thus preventing spasms and tetanus, and rendering any inflammation which may follow comparatively mild.

In simple fractures, and in the greater number of those which are complicated with displacement inwards, backwards, or outwards, with infiltration of blood, or laceration of the lateral ligaments, the apparatus is to remain applied from 25 to 35 days ; those which are complicated with serious injury of the soft parts, internal as well as external, with spiculæ, inflammation, suppuration, &c. will require from 40 to 60 days, and from 60, 80, to 100 days if the bones are much comminuted, and there is consequent necrosis of the bones or sloughing of the tendons. Convalescence is not, in general, completely established in less than double the time occupied in the actual treatment, whatever may be the kind of fracture. In all cases, on taking off the apparatus, the foot appears to be bent inwards, that is, in adduction. But the action of the muscles, or, in some cases, the application of the apparatus to the outside of the leg, speedily brings it to its natural position. In 207 cases treated thus by M. Dupuytren, 202 have been cured, 5 only have died, 2 of whom were destroyed by diseases independent of the injury. In all, the form of the limb has been restored, except two, in whom the heel has remained a little elongated backwards, whilst the tibia projected slightly forwards. All, except one, have recovered the free motions of the joint.

### Art. IX.—On False Aneurisms of the Brachial Artery.

Bleeding is often looked upon as too simple an operation to merit particular attention ; hence the comparative frequency of inflammation, phlebitis, varicose, diffused, and circumscribed aneurisms, following this small incision. During 15 years, M. Dupuytren, in his own practice, has had at least two cases of false aneurisms annually from this cause. These might all have been prevented by these few simple precautions;—1, Bleeding should never be performed before the pulsations of the artery have been felt. 2, The vein placed before the artery should *never* be opened. 3, Other veins should always be chosen, and, when they are not evident in the fold of the arm, recourse should be had to those of the fore-arm or hand. In the treatment of false aneurisms of the brachial artery, from puncture, M. Dupuytren employs one ligature above the tumour ; this treatment is almost constantly successful when the injury is recent, as the operation then places the lips of the wound of the artery in the condition of a fresh wound, disposed to unite; whilst the ligature has less chance of success when the puncture is of an older date, as its edges, being cicatrized, are incapable of adhesive inflammation. When a wounded artery is situated at the extremity of a limb, its communications are so numerous that it will require two ligatures. The particulars of four cases of false aneurism, consecutive to bleeding, are given; in all of them, M. Dupuytren tied the brachial artery, with one ligature, above the tumour, with success. In one of these the operation was performed two months after the injury, in another nine days after, in the third the time is not mentioned, but the original wound had healed, and the tumour was as large as the fist, and, in the last, the ligature was successful one month after the awkward venesection. Another case is detailed, in which two ligatures were applied to the radial artery, one above and the other below a false aneurism, produced by a wound by a penknife.

### Art. X.—On Fractures of the Patella.

Vertical fractures of the patella are not so uncommon as is imagined. Lamotte is the only French writer who has, perhaps, detailed a case with any precision. The fracture was the result of a fall ; the two portions of bone were slightly separated from each other, although the limb was demi-flexed. It was placed in an extended position, and the knee was enveloped with resolutive compresses, and a moderately compressing apparatus. Complete consolidation took place in 20 days. The callus was but slightly apparent.

Nearly 20 years ago, M. Dupuytren admitted into the Hôtel Dieu, a middle-aged man, who had fallen from a great height ; many bones were fractured, and the right knee was enormously bruised and deformed. The patient died on the third day. A longitudinal fracture of the patella was found, dividing the bone into two almost equal parts, the fragments were very moveable, there was manifest crepitation, and the capsule contained much bloody serum. Six months after a man was brought in, who was thrown down and run over by a carriage ; the wheel had passed over his leg from above downwards, and had fractured his patella in a longitudinal direction. Crepitation, as well as the displacement of the fragments, was evident, and the suitable apparatus was applied. He was going on favourably, when sesevere pneumonia proved fatal, 20 days after the accident. The parts were examined—a well-formed callus was found uniting the fragments, and scarcely permitting any motion ; the relation between the two surfaces was exact, and every thing announced a speedy and perfect cure. Three years after, a man entered the hospital on account of an ulcer on his leg. M. Dupuytren observed that the patella of one side was larger than the other, having a very apparent vertical ridge. On relaxing the extensors and moving the bone, this elevation was felt to rub against the condyles of the femur. On questioning him, he said that, some years previously, he had fallen and broken the leg in many places. It was clear that the patella had been vertically fractured. Another case, which terminated fatally, is given. The patient was a young woman, in very bad health at the time of the accident.

These fractures, says M. Dupuytren, depend always on the direct action of external causes, and are generally accompanied by wounds and contusions. The principles by which M. Dupuytren is guided in the treatment of transverse fractures of the patella are similar to those which are in vogue in this country, and his modifications of the apparatus recommended by Sir A. Cooper are too slight to require here a detailed explanation. He supports the leg in an extended position, with the heel higher than the pelvis, by numerous large pillows, extending from the foot to the tuberosity of the ischium; and he applies the bandages, which fix the two vertical compresses employed for drawing the broken portions of bone together, to the whole length of the foot and thigh. He admits that, unless the patient seconds the efforts of the surgeon, these means are insufficient to insure a close union. M. Dupuytren is convinced, from a great number of cases, that osseous union of the patella may take place, if the parts are retained in perfect contact during the necessary time. In ordinary cases, he agrees with Sir A. Cooper, that the union is cellulo-fibrous, but he shewed to our celebrated countryman, when he visited Paris in 1829, specimens where immediate union had taken place, and where this fibro-cartilaginous matter could not be perceived.

In vertical fractures, repose, immobility, and complete relaxation of the muscles are necessary. The limb should be a little elevated by pillows, and protected by a cradle from the pressure of the bed-clothes.

In fractures of the patella, as in those of the neck of the femur, consolidation of the callus takes place in from 60 to 80 days. After this period, if the state of the patient permits it, he may take slight exercise, as there is then no fear of the elongation of the callus; a knee-cap or simple bandage should be previously applied. Experience teaches that an osseous union may be obtained by a longer sojourn in bed; in a word, that the extent of separation, after the formation of callus, is in an inverse ratio to the prolongation of this confinement. In proof of this, a case is given of comminuted fracture of the patella, with other injuries, which forced the patient to remain in bed five months. The usual treatment was adopted, but, at the end of that time, the patella was so solidly united, that no traces remained of the solution of continuity; some small and very hard inequalities were alone felt on the surface.

### Art. XI.—General Considerations on the Treatment of Fractures of the Extremities.

In fractures of the upper extremities, without wounds, M. Dupuytren employs the roller; he places some compresses across the limb, opposite the fracture, besides the splints. In fractures of the humerus he uses many compresses, which he fixes with a few turns of the roller; over these he places four splints. In fractures of the fore-arm he pushes the flexor and extensor muscles into the inter-osseous space; over this he places graduated compresses and two splints; the bandage is applied from the fingers to the elbow. In fracture of the radius, besides the usual apparatus, he employs what he calls a cubital splint; it is made of iron, and curved at its inferior extremity; in the concavity of this curve are several buttons. The superior extremity of this metallic rod is fixed against the cubital border of the fore-arm. Between the inner border of the wrist and the convexity of the splint, a compress, folded many times, is placed to separate the one from the other, the hand is then fixed to the splint by means of a quilted compress, placed on its radial border between the thumb and the indicator, having two silk ribbons attached to its two extremities, which are to be tied to the cubital splint, and prevented from slipping by its buttons. In fractures of the olecranon, he places compresses only above the fracture; his anterior splint is straight. In fractures of the lower extremities, where the roller is so liable to be displaced, he employs the bandage of Scultetus.

### ART. XII.—EXCISION OF HÆMORRHOIDAL TUMOURS.

M. Dupuytren agrees with Stahl, Morgagni, Petit, &c. that hæmorrhoidal tumours are dilated veins. They are either external or internal. When internal, they are covered with mucous membrane of a violet colour, forming a partition across the rectum divided by furrows, which are sometimes obliterated by inflammation. These are venous dilatations like the heads of pins, in the tissue of the mucous membrane itself, from which venous blood flows when they are cut, giving the membrane a spongy appearance. Beneath the mucous coat are false organized membranes, or the cellular coat, and next the muscular. Large arteries often accompany closely the dilated veins. The external tumours, which form a sort of corona around the anus, are composed—1, Externally, in great part by the rectum, and a little by the skin; 2, False membranes, or the nervous coat, which then seems to be continuous with the fascia superficialis; 3, The dilated veins, which constitute the piles; 4. The external sphincter which embraces their pedicle, and constantly covers them partially with its fibres; 5, Nervous filaments; 6, Fat, which is sometimes placed between the skin and these tumours. No attempt should be made to cure these tumours in individuals weakened by organic disease, of the liver, intestines, or especially of the lung. The symptoms of phthisis have been suspended for a considerable time by the presence of piles, and have re-commenced with all their destructive energy after the unadvised suppression of the evacuation. In women far advanced in pregnancy, or after accouchement, piles are not infrequent; they disappear with the evident cause. Surgical assistance is not necessary when the hæmorrhoids are not degenerated in their structure, and when they do not give rise to hæmorrhages and abundant purulent discharges which throw the patients into a profound and characteristic anemia; for the symptoms are then relieved by antiphlogistics. But when they are degenerated and give rise to these symptoms, excision alone, according to M. Dupuytren, gives any chance of success.

The external hæmorrhoidal tumours form a circular row of smooth rounded tubercles, of a brownish colour externally, where they are covered with skin, and of a vivid red internally, where the mucous membrane covers them; their external surface is rarely ulcerated, their internal frequently giving rise to hæmorrhages and purulent effusions, which tend to debilitate the patient. The internal tumours are often strangled by the sphincters, owing to an engorgement or prolapsus of the mucous membrane—a frequent complication in piles. They give rise to the same symptoms as the external, and form tubercles of a vivid red colour. Both kinds may be present in the same patient. Individuals affected with this complaint walk with difficulty; they are constantly compelled to stop from the acuteness of their pain; they endeavour, by sitting down, by rubbing against a wall, or by their hands to return the tumours which have protruded, but the relief is momentary, and the return of pain follows a fresh prolapse. More or less weakened by hæmorrhage or sero-purulent discharge, their skin becomes pale, colourless, dull, and wax-like; they become sad and melancholy, their intellects are weakened, and they often attempt suicide. The local degeneration goes on, scirrhus attacks the anus and lower part of the rectum, and death would be the effect of this, as well as of the abundant discharge, if the progress of the disease was not effectually opposed by their removal. The question is, what is the best mode? Compression may obliterate such tumours, but the situation in this case prevents its application. Ligature exposes the patient to inflammation, insupportable pain, and sometimes to death. Cauterization is of incontestible service when it follows excision, but when applied to large tumours it produces excessive pain, and it may be productive of danger. Recision, or opening the veins with scissors, produces hæmorrhage and inflammation, whilst it leaves the tumours. Excision is the preferable operation, and M. Dupuytren employs it with the greatest success. The patient is directed to lie on his side on the edge of the bed, or on his elbows and knees, his legs being extended; or, what is better, one leg bent strongly on the thigh, and the other extended. If the tumour is internal, the patient is recommended to force violently as if he were at stool; an assistant separates the buttocks, whilst the operator seizes the tumour with a pair of broad forceps, and with a pair of long-curved scissors removes it. Only that portion of the tumour projecting outwards should be cut off; for if the whole is taken away, the patient will be exposed to serious hæmorrhage, and a consecutive contraction of the anus. In acting thus, a considerable mass is left at the margin of the anus, so that a sufficient quantity does not appear to have been taken away; but when cicatrization is completed, all returns to a normal state. The same takes place in excision of the tonsils. The excision of internal piles is less easy; the patient should sit over warm water, and make great expulsive efforts; he then should immediately lie in bed in the position recommended, so that the operator may at once seize and remove the protruded tumour before it re-enters. A laxative and a glyster should be given previously. The danger of excision wholly depends on the hæmorrhage which may follow. This is to be stopped by the actual cautery. When the tumour is external the operation is without difficulty; when internal it is not so easy, and the hæmorrhage is not so simply detected. The symptoms of the internal hæmorrhage are, a feeling of heat in the belly, which gradually extends upwards as the blood accumulates; or colic and tenesmus. Sensibility about the lower part of the abdomen, especially towards the left iliac fossæ; respiration difficult and interrupted; the pulse, at first intermittent and irregular, becomes small and frequent; the skin is colourless, and the face is covered with cold sweat. Anxiety, despair of recovery, nausea, vomiting, convulsions, vertigo, &c. As soon as this is recognized, the patient should be directed to expel the blood, and a cold lavement should be given; the efforts expose the wound, and that part from which a jet of blood comes, is to be touched with the cautery heated to a white heat. M. Dupuytren has had instruments constructed for this purpose, one like a bean, and another cylindrical, (cautères en haricot, et en roseau.) This always stops the blood, and M. Dupuytren has never seen it followed by any danger. Indeed, whenever he removes piles, he leaves with the patient an intelligent assistant, with directions to use the cautery on the first indications of external or internal hæmorrhage. A less certain way of producing the same effect is the introduction of a pig's bladder into the anus, and filling it with charpie. This succeeded in one case in which M. Dupuytren tried it, but it is very inconvenient to the patient, and is almost always expelled by the involuntary contraction of the parts which it causes. A considerable tumefaction of the cellular and adipose tissue of the anus follows excision, producing irritation of the rectum, and impossibility of going to stool for four or five days. The preceding measures, and the low diet in which they are kept subsequently to the operation, prevent any bad effects arising from constipation. The tumefaction yields readily to leeches, fomentations, &c. The sudden removal of the cause of such large evacuations in those who have been reduced to a state of anemia, often produces artificial plethora, and sanguineous congestions of the various organs. If the patient is young and vigorous, and the discharge was sanguineous, he must be repeatedly bled, at short intervals; in opposite circumstances, another discharge must be substituted, as setons or issues with laxatives. The cicatrization of the wound produced by the removal of an external hæmorrhoidal tumour, generally is sufficient to oppose the exit of the internal, so that the excision of the former is sufficient. If a second removal is practised, the tumour does not generally return, and the patient is cured for ever of his infirmity. Permanent contraction of the anus which has followed excision is prevented by the introduction of pledgets of linen into the anus, and renewing them until the wound has healed. Seven cases are detailed, illustrating these practical remarks of M. Dupuytren. In two the hæmorrhage was stopped by the bladder and charpie. In two, the cautery was applied immediately after excision; in two others it was used to stop internal hæmorrhage; in one only was excision alone sufficient. M. Dupuytren calculates from a large number of similar operations, both in hospital and private practice, that two-fifths of those who are not cauterized experience internal hæmorrhage, whilst it has never followed the application of the cautery. As, therefore, it is impossible to tell, à priori, if the patient will be among the small number of those that escape, and as the sequelæ of cauterization are not dangerous, but yield readily to simple means, should not the cautery be used always immediately after the operation? Such reasoning, together with the knowledge of the dangerous results of hæmorrhage, and the impossibility, in some cases, of rendering immediate and successful personal attention, M. Dupuytren acknowledges to be of weight, and such as to justify a modification of the operation he usually performs.

Here we must at present conclude; and if the first article on these volumes has occupied more room than was first intended, it must be attributed to the fund of useful and original matter which the work contains, and which rendered a shorter yet comprehensive analysis almost impossible. The persevering zeal and unwearied assiduity with which M. Dupuytren performs his public duties as a hospital surgeon and clinical professor, all who have attended the Hôtel Dieu will allow; and it would be unnecessary even for foreigners to laud his acknowledged talents and acquirements. We are therefore surprized at the bad taste which the physicians who have edited these volumes have exhibited in the profuse expenditure of common-place laudatory epithets, whenever the name, the talents, the experience, the lectures, the theories, the discoveries of M. Dupuytren are mentioned.

